# Why Are the Right and Left Hemisphere Conceptual Representations Different?

**DOI:** 10.1155/2014/603134

**Published:** 2014-01-23

**Authors:** Guido Gainotti

**Affiliations:** ^1^Center for Neuropsychological Research, Institute of Neurology Policlinico Gemelli, Catholic University of Rome, Largo A. Gemelli 8, 00168 Rome, Italy; ^2^IRCCS Fondazione Santa Lucia, Department of Clinical and Behavioral Neurology, Via Ardeatina 306, 00179 Rome, Italy

## Abstract

The present survey develops a previous position paper, in which I suggested that the multimodal semantic impairment observed in advanced stages of semantic dementia is due to the joint disruption of pictorial and verbal representations, subtended by the right and left anterior temporal lobes, rather than to the loss of a unitary, amodal semantic system. The main goals of the present review are (a) to survey a larger set of data, in order to confirm the differences in conceptual representations at the level of the right and left hemispheres, (b) to examine if language-mediated information plays a greater role in left hemisphere semantic knowledge than sensory-motor information in right hemisphere conceptual knowledge, and (c) to discuss the models that could explain both the differences in conceptual representations at the hemispheric level and the prevalence of the left hemisphere language-mediated semantic knowledge over the right hemisphere perceptually based conceptual representations.

## 1. Introduction

The construct of “semantic hub” has been proposed by Patterson et al. [1] and Lambon Ralph and Patterson [2] to identify a neural network, bilaterally supported by regions of the anterior temporal lobes (ATL), that should sustain the interactive activation of representations in all modalities and for all semantic categories. This construct was prompted by the observation of the selective loss of conceptual knowledge that can be observed in semantic dementia (SD) but has raised several empirical and theoretical objections. In a previous position paper [3], I have tried to evaluate if defects for items presented in every modality and across categories in SD necessarily point to an abstract-amodal format of the conceptual representations that are disrupted in this disease, or if alternative interpretations can be advanced. This problem is theoretically relevant, because the assumption of an abstract-amodal format of the conceptual representations is in keeping with cognitive models (e.g., [4–7]) that assume that semantic representations, accessed through structural descriptions, are stored in an abstract and propositional format. However, other models (e.g., [8–12]) refute the hypothesis of a unitary, abstract, and amodal semantic system and rather assume that conceptual knowledge is represented in the same format in which it has been constructed by the sensory-motor system. My conclusion, with respect to the abstract-amodal format of the conceptual representations disrupted in SD, counters a unitary system for two main reasons. The first was that the semantic impairment is “multimodal” only in the moderate to advanced stages of the disease, when atrophy affects the ATL bilaterally, whereas it can be modality specific in its early stages, when important asymmetries can be observed at the level of the ATL. In these cases, the loss of conceptual representations mainly affects the lexical-semantic knowledge when the left temporal lobe is more atrophic and the pictorial representations when the atrophy is greater on the right side. The second reason was represented by other clinical and neuroimaging data suggesting a prevalent involvement of the left and right ATL, respectively, in the processing of verbal and of pictorial data. I, therefore, concluded that the multimodal semantic impairment observed in advanced stages of SD is due to the joint disruption of pictorial and verbal representations, rather than to the loss of amodal knowledge, bilaterally supported by the ATL. In my analysis of papers relevant to the issue of the relationships between right or left prevalence of the ATL atrophy and level of impairment shown on verbal or pictorial task of semantic knowledge (or between side of the prevalent ATL activation and verbal or nonverbal nature of the stimuli) I noticed that an imbalance existed between the greater weight of verbally coded information in the left ATL and that of the nonverbal information in the right ATL. As a matter of fact, in cases of semantic dementia with structural neuroimages visualizing asymmetric atrophies, the loss of lexical-semantic knowledge when the atrophy was greater on the left side was more striking than the loss of nonverbal representations when the right temporal lobe was more atrophic. The reciprocal finding was observed in functional neuroimaging experiments, where the activation with verbal stimuli observed in the left hemisphere was more extensive and severe than the activation observed in the right hemisphere with nonverbal stimuli. These observations did not undermine my claim that the format of conceptual representation is different at the level of the right and left ATL (and in general of the right and left hemisphere) but raised the need to clarify the mechanisms underlying the greater representation of semantic-lexical knowledge at the level of the left hemisphere and of nonverbal (perceptually based) knowledge at the level of the right hemisphere. They, indeed, suggested that the difference between the (verbal) representations of semantic knowledge subsumed by the left ATL and the (sensory-motor) conceptual representations subtended by the right ATL is more quantitative than qualitative. If it was qualitative, as has been proposed, for example, by Paivio's [13, 14] “dual code” theory, visual and verbal information should be processed differently and along distinct channels in the human mind, creating separate representations for information processed in the right and left hemisphere. Therefore, there should be no prevalence of the left hemisphere verbal semantic knowledge over the right hemisphere perceptually-based conceptual knowledge, because verbal and pictorial information should be separately processed in (and be independently disrupted by pathology affecting) each hemisphere. On the other hand, a quantitative difference between the left verbal and the right perceptual components of the conceptual representations could be easily explained by the sensory-motor model of conceptual knowledge (e.g., [12, 15–20]). This model posits that each conceptual representation results from the convergence, in a “high order convergence zone” (Damasio, [21, 22]) of different perceptual, motor, and (verbally coded) encyclopaedic sources of knowledge.

It also posits that each of these sources of knowledge might have a different weight at the level of the right and left hemisphere and in the construction of different conceptual categories. According to this model, it could be logical to assume that in the left ATL there is large prevalence of language-mediated (over the sensory-motor) sources of knowledge, whereas the opposite (but milder) asymmetry could be found in the right ATL. de Renzi et al. (e.g., [23–29]) have, indeed, repeatedly shown that, even if sensory-motor information are bilaterally processed, the right hemisphere plays a greater role in this processing.

The present review will, therefore, have three different aims.

The first aim will consist in taking into account data surveyed in my previous paper [3], together with other data gathered from the literature, to confirm the different formats of conceptual representations at the level of the right and left ATL and, more in general, of the right and left hemispheres. The second aim will examine if language-mediated information plays a greater role in left hemisphere semantic knowledge than sensory-motor information in the right hemisphere conceptual knowledge. The last aim will discuss the models that could explain both the different (verbal and sensory-motor) formats of conceptual representations at the level of the left and right hemisphere and the prevalence of the left hemisphere verbal semantic knowledge over the right hemisphere perceptually-based conceptual representations.

To accomplish these aims, I will focus attention in the first part of my review on experimental studies which have investigated the relationships between results obtained from tasks using verbal or pictorial material and the right or left cerebral hemisphere, both in patients with unilateral brain damage and in normal subjects. Subsequently, I will review data from studies that contrasted performances obtained by SD patients with a greater degree of right or left ATL atrophy. Finally, I will review studies that evaluated the presence and severity of semantic-lexical disorders in right brain-damaged patients, to show that language participates to the composition of the right hemisphere conceptual representations, though to a lesser extent than to the construction of the left hemisphere semantic knowledge.

## 2. Methods

First, I reviewed all the clinical and experimental studies found in the neuropsychological literature that investigated the prevalence of verbal and nonverbal conceptual representations at the level of the right and left hemisphere. I required these studies to meet one of two criteria: (1) to be neuropsychological studies, which had compared memory or conceptual disorders observed with pictorial and verbal material in right and left brain damaged patients or (2) to consist of experiments conducted with pictorial and verbal material, to test the semantic capabilities of the left and right hemisphere in normal subjects. Secondly, I restricted attention to investigations which contrasted performances obtained on verbal and nonverbal conceptual tasks by semantic dementia (SD) patients with a prevalence of right or left anterior temporal lobe (ATL) atrophy. In the last section, I considered studies of patients with unilateral brain-damage that used tasks devised to check if language disorders of right brain-damaged patients selectively affect the semantic-lexical level.

In the selection of studies to be included in the first two sections I considered as “verbal” only tasks based on purely linguistic material and as “pictorial” only tasks based on purely “nonverbal” material, excluding picture naming and word-to-picture matching tasks, because these tasks involve both verbal and nonverbal materials. The only exception to this rule was represented by the study of Mion et al. [30] that had investigated the neural correlates of verbal and nonverbal conceptual processing in SD, using a picture naming task to assess verbal semantic memory. Since this study had explicitly tested the aims of our survey, we argued that to exclude this study from our review because the semantic verbal task was only partially appropriate would be excessive.

A different criterion was used in the last section of our review, because in this case our scope was not to compare the verbal versus pictorial representation of concepts in the left versus right hemisphere, but to check if language disorders of right brain-damaged patients selectively affect the semantic-lexical level. Being the scope of this section different from that of the first two sections, we reasoned that all kinds of semantic tasks, irrespectively of their verbal or nonverbal nature, were appropriate to check this issue.

## 3. Results of Investigations Which Have Compared Memory or Conceptual Disorders Observed with Pictorial and Verbal Material in Right and Left Brain-Damaged Patients and of Neuroimaging Experiments That Were Conducted to Assess the Semantic Capabilities of the Left and Right Hemisphere in Normal Subjects

Table 1 reports the results of behavioural studies, neuroimaging investigations, and TMS experiments, which have compared memory or conceptual disorders observed with pictorial and verbal material in right and left brain-damaged patients or assessed the semantic capabilities of the left and right hemisphere in normal subjects.

Results of behavioural studies have shown that, in investigations conducted on right and left brain-damaged patients, irrespectively of the procedures used (two forced-choice recognition memory in the study of Whitehouse [31]; categorization of perceptual and conceptual stimuli in the experiment of Grossman and Wilson [32] or administration of verbal and pictorial memory tasks in the study of Gainotti et al. [33]), left hemisphere injuries selectively impair the verbal memory code, whereas right brain damage preferentially undermines the pictorial code. Analogous results were obtained in experiments conducted in normal subjects, studying the categorization of verbal and pictorial stimuli tachistoscopically presented to the right and left hemisphere [34]. Similar results have also been found in studies using a semantic priming paradigm to examine whether perceptual or conceptual components of word meanings would be associated with the left or right hemisphere [35]. Results of neuroimaging investigations in normal subjects and brain-damaged patients are consistent with these behavioural studies. In two activation studies using verbal and nonverbal stimuli, each administered in the visual and auditory modalities, Thierry et al. [36] and Thierry and Price [37] found that the left temporal regions were more involved in the comprehension of verbal stimuli, whereas the right temporal cortex was more involved in understanding the meaning of environmental sounds and images. These results were confirmed by Hocking and Price [38], who showed that matching of verbal (visual and auditory) stimuli increased activation in the left temporal cortex, whereas matching of nonverbal visual and auditory stimuli increased activation in the right fusiform cortex. Similar results were obtained by Acres et al. [39] and by Butler et al. [40], who used voxel-based lesion-symptom mapping to correlate performance on verbal and nonverbal semantic tasks with involvement of the right and left temporal lobes. They reported material-specific correlations greater for verbal stimuli with left temporal regions than for nonverbal stimuli with the right fusiform gyrus. At variance with all these data were results obtained by Pobric et al. [41], using offline, low-frequency, repetitive transcranial magnetic stimulation (rTMS) to temporarily disrupt neural processing of verbal and nonverbal stimuli in the left or right temporal poles. During the induced refractory period rTMS applied to the left or right temporal poles disrupted semantic processing for words and pictures to the same degree. Thus, with the exception of these rTMS data, results reported in Table 1 show a prevalent involvement of the left and right hemispheres, respectively in the processing of verbal and of pictorial data, substantially confirming the different format of conceptual representations at the level of the right and left hemisphere.

It must also be acknowledged that other data supporting the hypothesis of a greater involvement of the left hemisphere in semantic verbal processing and of the right hemisphere in the processing of pictorial data have not been taken into account in the present review because of our strict selection criteria and of the decision of excluding from the survey results that could be considered as questionable. For example, we have not reported in Table 1 a study of Vandenberghe et al. [42] which is generally cited as in accordance to an amodal system, even if the data of this study were subsequently reanalyzed by Thierry and Price [37], showing a prevalent activation of the left hemisphere for verbal and of the right hemisphere for nonverbal material.

Regarding a related phenomenon, that of dual coding of nameable pictorial material, data reported in Table 1 also confirm the greater weight of verbally coded information in the left hemisphere in comparison to that of the nonverbal material in the right hemisphere. For instance, in the Nieto et al. [34] study, a right visual field advantage was obtained for a categorization task using verbal material, whereas the visual field differences were not significant when pictorial material was used. In a similar manner, in the Gainotti et al. [33] study, only the prevalent disruption of verbal memory in left BD patients was significant, whereas the trend in the opposite direction shown by right BD patients on the test of pictorial memory was nonsignificant. In Thierry et al. [36, Figure 3] functional imaging study, the selective left temporal lobe (TL) activation when comprehending words was more extensive than the right TL activation when processing nonverbal sounds. Finally, also in the Acres et al. [39, Figure 2] study of the neural correlates of scores obtained on the picture versus word categorization tasks and in the Butler et al. [40, Figure 1] comparison between the neural correlates of performance obtained on the verbal and the pictorial version of the Pyramid and Palm Trees Test (PPT [48]), the correlations between measures of neuronal integrity and performance on verbal and nonverbal semantic tasks were stronger for verbal stimuli in left temporal regions than for nonverbal material in the right inferior temporal cortex.

## 4. Results Obtained in Investigations Which Have Contrasted Performances Obtained on Verbal and Nonverbal Semantic Tasks by SD Patients with a Prevalent Right or Left ATL Atrophy

Table 2 reports the results of behavioural, neuropsychological, and neurophysiological investigations, which have contrasted performances or anatomoclinical correlations of results obtained on verbal and nonverbal semantic tasks by SD patients with a prevalent right or left ATL atrophy.

The anatomoclinical correlates of performances obtained on verbal and nonverbal conceptual tasks by SD patients with a prevalent atrophy of the right and left ATLs also demonstrated a lateralized specialization in semantic processing [30, 43, 47]. Snowden et al. [43, 47] contrasted results obtained by SD patients, whose temporal lobe atrophy was more severe on the left or on the right side, on the recognition of famous faces and names and on the verbal and pictorial versions of the PPT. Mion et al. [30] examined the neural correlates of verbal and nonverbal semantic measures in SD using FDG-PET. The verbal semantic task was a picture naming task, whereas the nonverbal semantic task was a pictorial version of the “Camel and Cactus test” [49]. Despite the differences in methods and materials used, results were very similar. In the Snowden et al. [43] study, subjects with a predominance of left TL atrophy identified faces better than names, whereas patients with a more severe right TL degeneration showed the opposite pattern. Furthermore, a better identification of famous faces was associated with superior performance on the picture, compared with the word, versions of the PPT, suggesting that objects and unique entities are represented in the same (pictorial versus verbal) format in the right and left temporal lobes. These results were confirmed by the same authors [47] in a further study in which statistical comparisons were based on nonparametric performance rankings in which face and name identification significantly distinguished the two SD subgroups, whereas comparisons between picture and word versions of the PPT tests only approached significance. In the Mion et al. [30] study, in which regions of interest (ROIs) were the left and right anterior fusiform gyri and the temporal poles, the left anterior fusiform activity predicted performance on the verbal semantic tasks, whereas the right anterior fusiform metabolism predicted performance on the pictorial nonverbal semantic task. Furthermore, an additional behavioural study, performed on a wider cohort of SD patients, confirmed that patients with more extensive right TL atrophy are significantly more impaired on tests of nonverbal semantics.

Results of these anatomoclinical correlative studies were confirmed by those of a behavioural investigation [44] and of a neurophysiological study [45]. Ikeda et al. [44] administered to SD patients and matched controls an object recognition task in which they were invited to classify as the same thing two visually different pictures of the same object. The patients with a greater right temporal atrophy were more impaired than those with a predominantly left-sided atrophy, showing that they had a selective defect in the retrieval of the perceptual properties of objects. Hurley et al. [45] studied the abnormalities of the N400 component of the ERP in patients with atrophy of the right and left ATL in a picture-probe matching task in which probes were semantically related and unrelated words and pictures. SD patients' N400 was significant when viewing unrelated mismatches, but not in response to the related pairings. This blunting of intracategory distinctions in the N400 response was selectively correlated with atrophy of the left but not right anterior temporal lobe. This N400 abnormality was not present in a parallel nonverbal part of the experiment where object pictures were matched to other object pictures [46]. Taken together, these behavioural, anatomoclinical, and neurophysiological investigations confirm the prevalent involvement of the left and right ATLs in the processing of verbal and of pictorial data, respectively, and, therefore, the different formats of conceptual representations that in the previous section had been shown to be present at the level of the right and left hemispheres. At the same time, the greater weight of verbally coded information in the left ATL in comparison to that of the nonverbal material in the right ATL is confirmed by the Mion et al. [30, Figures 3 and 4] observation that the involvement of left temporal lobe in the verbal semantic tasks was greater than that of the right ATL in the nonverbal conceptual tasks.

## 5. Studies Which Have Tried to Evaluate Presence and Severity of Semantic-Lexical Disorders in Right Brain-Damaged Patients

I have assumed in the introductory section of this survey that conceptual representations result from the convergence in “high order convergence zones” [21, 22] of different perceptual, motor, and (verbally coded) encyclopaedic sources of knowledge. I have also presumed that each of these “sources of knowledge” may have a different weight both at the level of the right and left hemisphere and in the construction of different conceptual categories. If these postulates are correct, language should participate selectively in the composition of the right hemisphere conceptual representations, though to a lesser extent than in the construction of the left hemisphere semantic knowledge. In order to check this assumption, I have surveyed the (usually old) studies that sought to evaluate the presence and severity of semantic-lexical disorders in right brain-damaged patients. Results of these studies have been summarized in Table 3.

Data reported in Table 3 confirm that patients with damage circumscribed to the right hemisphere, when submitted to word-picture matching tasks, show a defect of language comprehension that selectively affects the semantic-lexical level. This statement holds for the Lesser [50] study, in which 3 word-picture matching tasks selectively assessing phonological, lexical, and syntactic discrimination were administered to right and left BD patients, because semantic errors were found not only in aphasic patients but also, though to a smaller extent, in right BD patients. Analogous results were obtained by Gainotti et al. [51], who administered to right BD patients and normal controls (NC) the “verbal sound and meaning discrimination test” [52], a word-pictures matching task, allowing semantic, phonemic, and unrelated types of errors. In this case too, the number of semantic errors obtained by right BD patients was higher than that obtained by NC, but lower than that previously obtained on the same test by aphasic LBD patients [52].

Very similar results were also obtained by Gainotti et al. [53], who administered to right BD patients and NC an aural and a written test of semantic discrimination and a test of phoneme discrimination. Right hemisphere lesions consistently impaired semantic-lexical discrimination in both the aural and the written modality, without affecting phoneme discrimination, but the number of errors obtained on the semantic tests by RBD patients was less than that obtained on the same tests by aphasic LBD patients [56]. It could be objected that on word-picture matching tasks errors could be due to the pictorial nature of the responses rather than to properly semantic-lexical difficulties, but very similar results have been obtained by Joanette and Goulet [54], who administered to RBD patients and NC a verbal fluency task for which acceptability criteria were either semantic or orthographic and by Neininger and Pulvermüller [55], who used a speeded lexical decision task to investigate word-category deficits in RBD patients and in NCs. In this latter study, patients with lesions in the right frontal lobe showed most severe deficits in processing action words, whereas those with right temporooccipital lesions showed most severe deficits in processing visually related nouns. According to Neininger and Pulvermüller [55], Hebbian correlation learning [57] implies that a word frequently cooccurring with a visual stimulus will be stored in the cortex by means of strong connections between neurons in visual and language areas. However, since neurons in both hemispheres are related to the execution of body movements and to the perception of objects, both action and visually related right hemisphere areas should serve as complementary, category-specific language-related areas for processing action words. According to this interpretation that is consistent with the positions advanced in previous sections of this survey, word processing is based on cell assemblies distributed over both hemispheres whose right hemispheric components constitute integral aspects of the right hemisphere semantic representation.

## 6. General Discussion

Results of the present survey seem to support the following statements.

(a) Conceptual representations have different formats at the level of the right and left hemispheres (Table 1) and, more specifically, at the level of the right and left ATLs (Table 2), being mainly based on sensory-motor information on the right side and on language-mediated sources of knowledge on the left side of the brain. These findings are inconsistent with cognitive models (e.g., [4–7]), which assume that semantic representations constitute a unitary system and are stored in an abstract and propositional format. They, rather, support the alternative models (e.g., [8–12, 18–20]), presuming that conceptual knowledge results from the convergence in “high order convergence zones” (e.g., [21, 22]), of different perceptual, motor, and (verbally coded) encyclopaedic sources of knowledge, that might have a different weight at the level of the right and left hemisphere.

(b) However, results of studies comparing memory or conceptual disorders using pictorial and verbal material in right and left brain-damaged patients (Table 1) or in patients with a prevalent right or left ATL atrophy (Table 2) have also shown that an imbalance exists between the greater weight of verbally coded information in the left hemisphere and that of the nonverbal information in the right hemisphere. All these studies have, indeed, shown that the loss of lexical-semantic knowledge is more striking when the lesions are on the left side than the loss of nonverbal representations when the right side is more damaged. These findings indicate that the difference between the (verbal) format of the semantic knowledge supported by the left hemisphere and the (sensory-motor) format of conceptual representations supported by the right hemisphere is not qualitative, as suggested by the Paivio (e.g., [13, 14]) “dual code” theory, but quantitative as proposed by the sensory-motor account of conceptual knowledge (e.g., [12, 15–20]). According to this model, it is, indeed, logical to expect a greater prevalence of verbally coded knowledge in the semantic representations of the language-dominant left hemisphere and a less striking prevalence of sensory-motor information in the conceptual representations of the right hemisphere. This is because, even if the right side of the brain plays a greater role in perceptual tasks (e.g., [23–29]), the processing of sensory-motor information is more strongly bilateral.

(c) Finally, data reported in Table 3 indicate that language participates to the composition of the right hemisphere conceptual representations, though to a lesser extent than to the construction of the left hemisphere semantic knowledge. A defect of language comprehension that selectively affects the semantic-lexical level has, indeed, been found in RBD patients with various kinds of word-picture matching tasks (e.g., [50, 51, 53]), verbal fluency tasks [54], and a speeded lexical decision task that aimed to investigate category-specific verbal deficits [55]. Results obtained by these last authors are particularly interesting from the vantage point of the present survey, because they show that convergence zones for different semantic categories can be found in different cortical areas (for action words in the frontal lobes and for visually related nouns in temporooccipital cortices) and that language participates in the composition of these semantic categories even in the right hemisphere.

Interpreting results of Neininger and Pulvermüller [55] in terms of the Hebbian correlation learning [57] could also be useful to account more generally for data gathered in the present survey. This interpretation implies that frequently cooccurring sensory-motor experiences (including the corresponding verbal components) will converge on strongly interconnected cell assemblies. Different cell assemblies should be localized in cortical areas involved in the treatment of the (visual, auditory, tactile, or action-related) sources of knowledge which play the most important role in the construction of different conceptual categories [55] and should be distributed on both hemispheres, but with a prevalent weight of the sensory-motor data in the right hemisphere and of the verbally-mediated information in the left hemisphere. The prevalence of verbally-mediated semantic knowledge in the left hemisphere does not require clarifications, whereas two (nonalternative) accounts could be offered to explain the prevalence in the right hemisphere of sensory-motor sources of knowledge. The first neuroanatomical interpretation has been proposed by de Renzi (e.g., [28, 29]), who has stressed the fact that the cortical areas involved in language processing in the left hemisphere could be (at least in part) dedicated to the sensory-motor processing in the homologous areas of the right hemisphere.

The second developmental explanation presumes that the “core properties” of concrete concepts may be built in the prelinguistic period, during our earliest experiences with objects, even though this primitive conceptual organization becomes very early intertwined with verbal processing (e.g., Friedrich and Friederici [58]). This interlacing between sensory-motor and verbal processing will strongly contribute to the conceptual development, due to the “catalytic and transformative” influence of language on cognition [59] and will allow the older children to move from a more perceptually based to a more linguistically based organization of the semantic system. Now, several lines of research (e.g., [60–63]) suggest that the maturation of the right hemisphere may precede that of the left hemisphere from both the structural and the functional point of view. From this vantage point, the prevalent sensory-motor format of conceptual representations at the level of the right hemisphere could be considered as a consequence of the “first come, first served” principle [61], according to which, the right and left hemispheres tend to remain linked in later stages of processing to the mechanisms in which they had played a dominant role in the earlier stages of the development.

The speculative nature of this general interpretation is obviously acknowledged, even if (a) the assumption that sources of knowledge experienced through diverse sensory modalities play different roles in the construction of different conceptual categories is consistent with the subjective evaluation of normal subjects (e.g., [64–66]) and (b) the prevalent involvement of the left ATL in verbal and of the right ATL in pictorial/sensory aspects of conceptual knowledge has been documented in previous papers (e.g., [3, 30, 43, 47]).

## Figures and Tables

**Table 1:** Results of neuropsychological investigations that have compared memory or conceptual disorders observed with pictorial and verbal material in right and left brain-damaged patients and of experiments conducted with similar material to test the semantic capabilities of the left and right hemisphere in normal subjects.

Authors	Methods	Results
(1) Behavioral studies in normal subjects and brain-damaged patients		
Whitehouse [31]	Explored in R and LBD patients aspects of pictorial and verbal encoding in two forced-choice recognition memory experiments.	Left hemisphere injury selectively impaired verbal memory coding, whereas right hemisphere damage preferentially impaired pictorial coding.

Grossman and Wilson [32]	Asked right and left BD patients and normal controls to evaluate perceptual and conceptual stimuli for their degree of category membership.	The left-hemisphere patients showed anomalies in categorizing the conceptual but not the perceptual items, while the reverse was true for the right hemisphere patients.

Nieto et al. [34]	Carried out two lateral tachistoscopic experiments in normal subjects, to test semantic capabilities of the left and right cerebral hemispheres, through categorization tasks with verbal and pictorial presentation.	Right visual field advantages were obtained for verbal presentations in both category-membership and category-matching tasks. However, no significant visual field differences were found for any pictorial presentations.

Gainotti et al. [33]	Constructed two very similar tasks of verbal and pictorial memory and administered them to control subjects and patients with R and L hemispheric lesions.	Word recognition was selectively impaired by left and picture recognition by right brain injury, but the difference between R and LBD patients was significant only on the test of verbal memory, whereas the trend in the opposite direction observed on the test of pictorial memory was nonsignificant.

Shibahara and Lucero-Wagoner [35]	Used a semantic priming paradigm to examine whether perceptual or conceptual properties of word meanings would be associated with the left or right hemisphere.	The results indicated that perceptual information is available only in the right hemisphere while conceptual information is available in both hemispheres.

(2) Neuroimaging investigations in normal subjects and brain-damaged patients		
Thierry et al. [36]	Used functional neuroimaging in normal subjects to compare semantic processing of spoken words to equivalent processing of environmental sounds, after controlling for low-level perceptual differences.	Words enhanced activation in left temporal (LT) regions while environmental sounds enhanced activation in the right temporal (RT) areas. The LT involvement in comprehending words was more extensive than the RT involvement in processing non-verbal sounds.

Thierry and Price [37]	Developed these studies, comparing conceptual processing of verbal and non-verbal stimuli in both visual and auditory modalities.	They found that left temporal regions were more involved in comprehending words (heard or read), whereas the right temporal cortex was more involved in making sense of environmental sounds and images.

Acres et al. [39]	Administered four verbal and non-verbal tasks (including words and pictures categorization) to patients with R and L temporal lesions and correlated their behavioural scores with voxel-based measures of neuronal integrity.	Performance on the verbal tasks correlated with the lesion of left inferior and anterior temporal regions, while performance on the non-verbal tasks correlated with the lesion of analogous right temporal areas. The L temporal lobe was more involved in word categorization than the right in pictures categorization.

Butler et al. [40]	Used voxel-based morphometry to correlate performance on verbal and nonverbal versions of a semantic association task in patients with neurodegenerative diseases.	They found material-specific correlations, greater for verbal stimuli in left temporal regions than for nonverbal stimuli in the right fusiform gyrus.

Hocking and Price [38]	Presented subjects simultaneously with one visual and one auditory stimulus and instructed them to decide whether these stimuli referred to the same object or not. Verbal stimuli consisted of spoken and written object names, whereas non-verbal stimuli consisted of pictures of objects and naturally occurring object sounds.	Verbal matching increased activation in the left temporal lobe, whereas non-verbal matching increased activation in the right fusiform region.

(3) TMS experiments in normal subjects		
Pobric et al. [41]	Used offline, low-frequency, and repetitive transcranial magnetic stimulation (rTMS) to disrupt neural processing temporarily in the left or right temporal poles. During the induced refractory period, subjects made judgements of semantic association for verbal and pictorial stimuli.	They found that rTMS applied to the left or right temporal poles disrupted semantic processing for words and pictures to the same degree.

R: right, L: left, BD: brain damaged.

**Table 2:** Data obtained in investigations which contrasted performances obtained on verbal and non-verbal semantic tasks by patients with semantic dementia (SD) with prevalent right or left anterior temporal lobe (ATL) atrophy.

Authors	Methods	Results
Snowden et al. [43]	Administered to 15 SD patients, whose temporal lobe atrophy was more severe on the left or on the right side, tests of famous faces and names and the verbal and pictorial versions of the Pyramids and Palm Trees test (PPT).	Subjects with a predominance of left TL atrophy identified faces better than names and obtained better results on the pictorial than on the verbal version of the PPT test, whereas patients with a more severe right TL degeneration showed the opposite pattern.

Ikeda et al. [44]	Investigated if semantic dementia patients with prevalent left or right atrophy have different impairments of object recognition, as measured by the ability to classify two visually different tokens of an object as the same thing.	The impairment was greater for cases whose anterior temporal lobe atrophy was prevalent on the right than for those with a prevalence of left-sided atrophy.

Mion et al. [30]	Examined in SD the neural correlates of verbal and non-verbal semantic measures with FDG-PET. The semantic verbal task was picture naming, whereas the non-verbal semantic task was a pictorial version of the “Camel and Cactus test”. The authors performed an additional behavioural study on a wider cohort of patients with semantic dementia.	The left anterior fusiform activity predicted performance on the verbal semantic task whereas the right anterior fusiform metabolism predicted performance on the perceptual semantic task. The L temporal lobe was more involved in the verbal semantic tasks than the right in the non-verbal task. Patients with more extensive right temporal atrophy were significantly more impaired on the test of non-verbal semantics.

Hurley et al. [45]	Studied the abnormalities of the N400 component of the ERP in a picture-probe matching task in patients with atrophy of the right and left ATL. Probes were semantically related and unrelated words and pictures.	A significant N400 potential was found in patients with atrophy of the left but not of the right ATL [46] only when the probe was a semantically unrelated but not a related word.

Snowden et al. [47]	Reexamined performance of SD patients with predominantly right and left TL atrophy, not only on faces and names of famous people, but also on the pictorial and word versions of the PPT test. They based statistical comparisons on individual performance ranks.	Differences in rankings for face and name identification strongly distinguished the two SD subgroups, whereas comparisons between picture and word versions of the PPT tests only approached significance.

ERP: event related potentials, TL: temporal lobe.

**Table 3:** Results of studies conducted on patients with unilateral brain-damage with tasks devised to test semantic-lexical disorders of right brain-damaged patients.

Authors	Methods	Results
Lesser [50]	Administered to R and LBD patients three word-picture matching tasks for a selective assessment of phonological, semantic, and syntactic comprehension.	They found that not only aphasic patients, but also, though to a smaller extent, RBD patients had a semantic comprehension deficit

Gainotti et al. [51]	Gave to RBD patients and normal controls a word-picture matching tasks (the Verbal Sound and Meaning Discrimination test) allowing semantic, phonemic and unrelated types of errors.	Patients with RBD obtained significantly more lexical-semantic errors than normal controls The number of semantic errors obtained by RBD patients was significantly lower than that obtained on the same tests by aphasic LBD patients [51].

Gainotti et al. [53]	Administered to RBD patients and normal controls two tests of semantic discrimination (auditory language comprehension and reading comprehension) and a test of phoneme discrimination.	Right hemisphere lesions consistently impaired semantic discrimination in the oral and written modality, but did not hamper phoneme discrimination. The number of errors obtained on the semantic tests by RBD patients was lower than that obtained on the same tests by aphasic LBD patients [53].

Joanette and Goulet [54]	Submitted RBD patients and control subjects to a verbal fluency task for which acceptability criteria were either semantic or formal.	Subjects with right hemisphere lesion showed a significant reduction of verbal fluency, as compared to controls, only when the criterion was semantic.

Neininger and Pulvermüller [55]	Used a speeded lexical decision task to investigate word-category deficits in patients suffering from lesions in the right hemisphere and in normal controls.	Patients with lesions in the right frontal lobe showed more severe deficits in processing action whereas those with lesions in their right temporo-occipital areas showed more severe deficits in processing visually related nouns.

R: right, L: left, BD: brain damaged.
